# Genetic Variability of Microcystin Biosynthesis Genes in *Planktothrix* as Elucidated from Samples Preserved by Heat Desiccation during Three Decades

**DOI:** 10.1371/journal.pone.0080177

**Published:** 2013-11-12

**Authors:** Veronika Ostermaier, Guntram Christiansen, Ferdinand Schanz, Rainer Kurmayer

**Affiliations:** 1 Research Institute for Limnology, University of Innsbruck, Mondsee, Austria; 2 Limnological Station, Institute of Plant Biology, University of Zürich, Kilchberg, Switzerland; University of New South Wales, Australia

## Abstract

Historic samples of phytoplankton can provide information on the abundance of the toxigenic genotypes of cyanobacteria in dependence on increased or decreased eutrophication. The analysis of a time-series from preserved phytoplankton samples by quantitative PCR (qPCR) extends observation periods considerably. The analysis of DNA from heat-desiccated samples by qPCR can be aggravated by point substitutions or the fragmentation of DNA introduced by the high temperature. In this study, we analyzed whether the heat desiccation of the cellular material of the cyanobacterium *Planktothrix* sp. introduced potential errors to the template DNA that is used for qPCR within (i) 16S rDNA and phycocyanin genes and (ii) the *mcy*A gene indicative of the incorporation of either dehydrobutyrine (Dhb) or N-methyl-dehydroalanine (Mdha) in position 7, and (ii) the *mcy*B gene, which is indicative of homotyrosine (Hty) in position 2 of the microcystin (MC) molecule. Due to high temperature desiccation, the deterioration of the DNA template quality was rather due to fragmentation than due to nucleotide substitutions. By using the heat-desiccated samples of Lake Zürich, Switzerland the abundance of the Dhb, Mdha and Hty genotypes was determined during three decades (1977-2008). Despite major changes in the trophic state of the lake resulting in a major increase of the total *Planktothrix* population density, the proportion of these genotypes encoding the synthesis of different MC congeners showed high stability. Nevertheless, a decline of the most abundant *mcy*A genotype indicative of the synthesis of Dhb in position 7 of the MC molecule was observed. This decline could be related to the gradual incline in the proportion of a mutant genotype carrying a 1.8kbp deletion of this gene region. The increase of this *mcy*A (Dhb) gene deletion mutant has been minor so far, however, and likely did not affect the overall toxicity of the population.

## Introduction

The microcystin (MC) synthetase (*mcy*) gene cluster encodes a mixed nonribosomal peptide synthetase (NRPS)/polyketide synthase (PKS) enzyme complex that produces the heptapeptide MC, which is the most frequent toxin occurring in blooms formed by cyanobacteria in freshwater. The *mcy* gene cluster has been sequenced from different cyanobacterial genera, e.g. *Microcystis*, *Planktothrix* and *Anabaena* [1-3]. NRPS are minimally composed of modules consisting of adenylation, thiolation, and condensation domains, which are responsible for the activation and condensation of amino acid substrates to the growing peptide chain. Additional domains regularly found in NRPS catalyze modification such as N-methylation (N-methylation domain, NMT), O-methylation, and the racemization of precursor amino acid substrates. The modular structure of NRPS has been linked to the high rate of evolution as gene collectives, as single genes or domains of different NRPS gene clusters can be horizontally exchanged and functionally interact to give rise to novel peptides (e.g. [4]). For *Microcystis*, the occurrence of different MC structural isoforms could be assigned to major genetic differences within the first adenylation (A1) -domain of *mcy*B [5], which were related to a recombination event between A-domains of *mcy*B and *mcy*C [6]. In *Planktothrix*, two distinct *mcy*A-A1-domain genotypes of the *mcy*A gene are known that differ in sequence and in the presence of a gene region encoding an N-methyltransferase domain [7]. It was concluded earlier that genetic recombination led to a complete replacement of a gene region coding for the first A-domain of McyA activating serine, which is later dehydrated to dehydroalanine (Dha). An integrated N-methyltransferase domain catalyzes the transfer of a methyl group to Dha resulting in N-methyl-dehydroalanine (Mdha) in position 7 of the MC molecule. Through recombination, a second genotype of the *mcy*A-A1-domain activates threonine, which is later dehydrated to dehydrobutyrine (Dhb) at the same position 7 of the MC molecule [7]. Further evidence for sequence variability and recombination events between the A-domains of *mcy*A, *mcy*B, and *mcy*C was given by Fewer et al. [8] and Tooming-Klunderud et al. [9]. 

The introduction of structural variability can have functional consequences, e.g. altered enzyme inhibition activity [10-13]. As evaluated by the *Thamnocephalus platyurus* toxicity assay, the toxicity of the MC variant with Dhb in position 7 ([D-Asp^3^, (*E*)-Dhb^7^]-MC-RR) was shown to be significantly higher compared to other MCs with Mdha in position 7. In contrast, the inhibition of the protein phosphatase 1 and 2A by the same MC variant was the lowest when compared with MC structural variants with Mdha in position 7 [11]. In mouse bioassays, the LD_50_ values of the MC-RR variants with Mdha in position 7 ([Asp^3^]-MC-RR) or Dhb in the same position ([Asp^3^, Dhb^7^]-MC-RR) were 500-800 and 250 µg kg^-1^ body weight, while the MC variant with homotyrosine in position 2 ([D-Asp^3^,(*E*)Dhb^7^]-MC-HtyR) showed a lower LD_50_ value of 70 µg kg^-1^ body weight [14,15].

While toxigenic cyanobacteria are routinely monitored by means of qPCR, e.g. [16], the quantification of individual genotypes encoding specific MC structural variants has received less attention. In a previous study, the proportion of genotypes indicative of Dhb or Mdha in position 7 or of homotyrosine (Hty) in position 2 of the MC molecule were compared among the populations of the cyanobacterium *Planktothrix* in lakes of the Alps over a period of two years (2005-2007). On average, the Dhb genotype was the most abundant and its abundance correlated with the higher concentration of the [Asp^3^, Dhb^7^]-MC-RR variant of the same samples [17]. Like other lakes in the Alps, Lake Zürich, Switzerland underwent major changes in its trophic state during the last 30 years and the population of red-pigmented *Planktothrix rubescens* increased from rare to a stable dominance of the plankton community [18,19]. During this re-oligotrophication phase, phytoplankton samples have been preserved by high temperature desiccation for the purpose of documentation. By utilizing these samples we showed that the proportion of toxigenic genotypes in total remained one hundred percent and genotypes that lost the *mcy* gene cluster [20] never became abundant [19]. However, in the previous study, the results on *mcy* genotype composition as well as the applicability of qPCR to preserved DNA samples have not been reported. As the samples from the last thirty years were preserved at 110°C for 2 h and stored at room temperature in the dark for decades, we were interested in the suitability of the DNA isolated from dried biomass for qPCR. Thus, the effect of high temperature desiccation on the applicability of the DNA for qPCR was evaluated experimentally. In order to examine the DNA damage from heat-desiccated samples, an enzyme cocktail formulated to reverse specific DNA base modifications (New England Biolabs, Germany) was applied. Finally, we aimed to correlate the *mcy* genotype abundance to the general increase in *Planktothrix* total population density, thus enabling conclusions on the success of certain *mcy*A or *mcy*B genotypes during 1977 to 2008. We analyzed the proportion of three different *mcy* genotypes that are indicative of Dhb or Mdha in position 7 or of Hty in position 2 of the MC molecule. Previously [21], a natural deletion mutant *mcy*HA (Dhb) carrying a 1.8 kbp deletion of the Dhb gene was described and this *mcy*HA mutant genotype would reduce the detectability of the Dhb genotype. In order to analyze the gradual decline of the proportion of the most abundant Dhb genotype, we used the data on the abundance of this *mcy*HA deletion genotype [19]. 

## Methods

### DNA isolation from heat-desiccated samples

As described previously, the DNA extracted from *Planktothrix* cells (and other phytoplankton) collected on filters from Lake Zürich (*n* = 111) during 1977-2008 has been used [19]. The phytoplankton biomass was heat-desiccated (110°C, 2 h) and stored at room temperature in the dark. For the high-temperature experiments, all DNA extractions were performed using a standard chloroform-phenol method [22]. 

### Experimental evaluation of high temperature-induced effects on DNA quality

Strains PCC 7821 (Dhb genotype) and No. 40 (Mdha genotype) were grown in BG11 medium [23] at 20°C under continuous light (5 to 15 μmol photons m^-2^ s^-1^). The cells were harvested on glass-fiber filters (BMC, Ederol, Vienna, Austria) during the exponential growth phase. The effect of heat desiccation on the DNA quality of the harvested cells was evaluated by means of qPCR using the 16S rDNA, Dhb, and Mdha gene loci (see below). Aliquots from cell cultures (PCC 7821: 107 ± 21 (SE) mm^3^ L^-1^, No. 40: 98 ± 26 mm^3^ L^-1^) were filtered and dried at three different temperatures (80°C, 110°C, and 150°C) for either 2 h or 12 h, each. As a control, one aliquot was frozen directly (-20°C). The experiment was repeated twice. 

### DNA treatment to reverse DNA base modifications

Since heat-induced base modifications resulting from the heat desiccation of DNA could not be excluded, the effect of an enzyme cocktail formulated to reverse DNA modifications (PreCR Repair Mix, New England Biolabs, Germany) was evaluated. The PreCR Repair Mix contains different enzymes for the repair of the most common lesions in DNA (abasic sites, nicks, thymidine dimers, blocked 3’ ends, oxidized guanine, oxidized pyrimidines, deaminated cytosine). In a first step, the efficiency of DNA treatment was evaluated by the application of the PreCR Repair Mix to aliquots of the DNA of a heat-preserved sample from 1980 under various incubations (37°C for 20 min, 4°C for 20 min, and 4°C overnight, respectively). A total volume of 25 µl included 1× ThermoPol buffer, dNTPs (100 µM final concentration), 1× NAD^+^, 0.5 µl of the Repair Mix and a final DNA amount of 125 ng. The treated DNA and aliquots of the untreated DNA were compared by qPCR using the 16S rDNA, Dhb, Mdha, and Hty gene loci (see below). No significant difference was found in the C_t_ values obtained by qPCR under the various incubation conditions (data not shown). Subsequently, all samples were incubated at 37°C (20 min). Aliquots of DNA isolated from four heat-desiccated samples from 1980, 1982, 1986, and 1988 were treated with the PreCR Repair Mix (37°C, 20 min). The efficiency of the PreCR Repair Mix was again tested by means of qPCR (see below). 

### Nucleotide variation among high temperature-desiccated samples

In order to find out whether high temperature caused a significant increase of DNA modifications within the gene loci to be analyzed by qPCR (16S rDNA, Dhb, Mdha, Hty), PCR products of a heat-desiccated sample (from 1980) were sequenced and then compared to sequences from PCR products obtained from a DNA sample stored frozen (from 2007). The PCR reactions were performed using Dream Taq polymerase (Thermo, St. Leon-Rot, Germany) according to the protocol of the manufacturer. For primer sequences, annealing temperatures, and the length of the PCR product, see Table S1 in File S1. PCR products were cloned and sequenced using vector primers according to standard procedures. The influence of the PreCR Repair Mix treatment was tested from aliquots by sequencing the 16S rDNA, Dhb and Hty gene locus (since the overall effect of PreCR treatment was rather small (see below) the Mdha gene locus was not sequenced after PreCR treatment). For comparing the variability within the 16S rDNA, Dhb, Mdha, and Hty genotype loci, the reference sequences of *Planktothrix* strains CCAP 1459/30 and No. 3 (16S: GQ994995.1, Dhb: AJ749260.1, Mdha: AJ749248.1, Hty: AJ749276.1) were used. 

### Quantification of toxigenic genotypes by qPCR

The Dhb and Mdha genotype, indicating the production of either Dhb or Mdha at position 7 and a genotype indicating Hty at position 2 of the MC molecule, were quantified by qPCR using the TaqMan assay. Primers and TaqMan probes were described previously [17,24]. For the quantification of genotypes, calibration curves were established by relating the dilution series of predetermined DNA concentrations (expressed in equivalents of cellular biovolume) from *Planktothrix* strains PCC 7821 (Dhb genotype) and strains No. 40 (Mdha genotype) and No. 21/1 (Hty genotype) to the measured C_t_ values as described [17,24]. The lowest dilution of the calibration series was defined as the limit of quantification, which corresponded to a four cells template^-1^. All of the samples were measured in triplicate on an Eppendorf Master Cycler Ep Realplex System (Eppendorf, Vienna, Austria) in a total volume of 25 µl. 

## Results

### Size range of DNA isolated from high-temperature desiccated samples

In order to estimate the degree of DNA fragmentation introduced by heat desiccation, the DNA was visualized by ethidium bromide staining using agarose gel electrophoresis. In general, the DNA extracted from the heat-desiccated samples of the years 1980, 1985, 1990, and 1995 was fragmented. Nevertheless, larger DNA fragments were visible (> 300 bp). Within the heat-desiccated samples, no correlation between the age of the isolated DNA and the grade of fragmentation was found (Figure S1 in File S1).

### Experimental evaluation of the high temperature induced effects on the qPCR results

**Figure 1:**
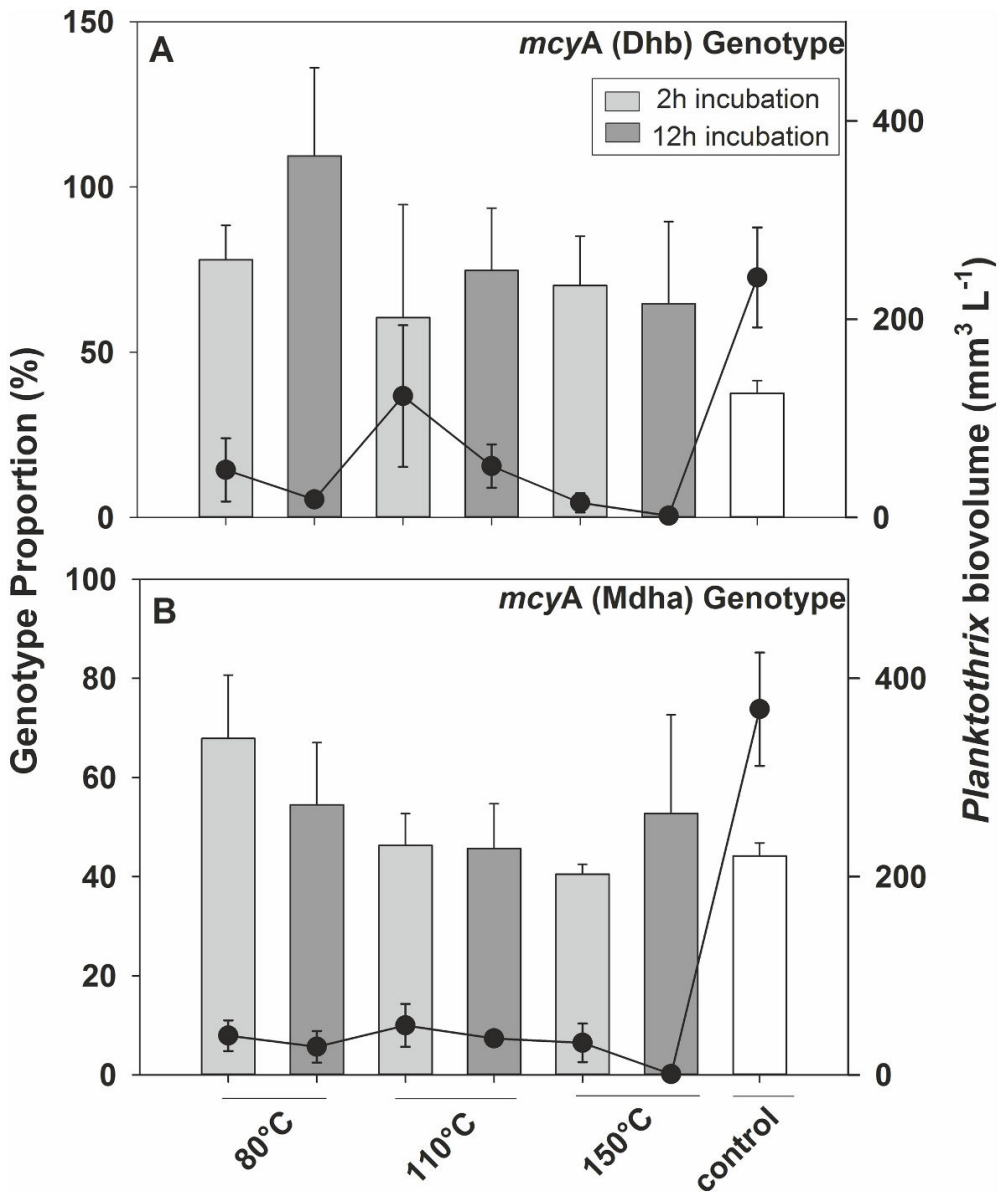
Average ± SE *mcy*A genotype biovolume (black line) and proportion (bars) estimated by means of qPCR from *Planktothrix* cell material preserved at different desiccation temperatures during 2 h (light grey bars) and 12 h (dark grey bars) of incubation. (A) Dhb genotype; (B) Mdha genotype.

Since heat-induced DNA modifications, such as nucleotide substitutions, could introduce a bias to the quantification of genotypes by means of qPCR, the DNA extracted from heat-desiccated samples was compared quantitatively to the DNA that was extracted from samples stored frozen. In general, through high temperature, a significant reduction in DNA template concentration (expressed in equivalents of cellular biovolume) occurred for all gene loci (One Way ANOVA, *p* < 0.01). For the 16S rDNA locus, the lowest DNA concentration was measured when the filters were dried at 150°C (12 h): 0.1-15% of the biovolume of the control (Figure 1). The duration of high temperature incubation significantly reduced the DNA template concentration further (pairwise *post-hoc* test, *p* < 0.05). Overall, the DNA template concentration of heat-desiccated filters of the strains PCC 7821 and No. 40 accounted for 18 ± 7% (min 0.4, max 120%) and 7 ± 2% (min 0.1, max 22%), respectively. 

For both the Dhb and Mdha genotype, the high temperature incubation led to increased proportions, but this preservation effect was not statistically significant (One Way ANOVA, Dhb: *p* = 0.12, Mdha: *p* = 0.4, Table S2 in File S1). In contrast to the heat-induced decrease in DNA template concentration, there was no significant difference between incubation times (2 h vs 12 h, Dhb: *p* = 0.33, Mdha: *p* = 0.84). Altogether, these results indicate that the average proportion of both the Dhb and Mdha genotype seemed to be relatively unbiased through high-temperature desiccation. 

### Evaluation of DNA treatment to reverse DNA modifications

Four randomly selected DNA samples were treated with the PreCR Repair Mix and the DNA template concentration as estimated by qPCR was compared quantitatively to untreated aliquots for four different loci (16Sr DNA, Dhb, Mdha, and Hty). In the majority of samples the difference in C_t_ values between treated DNA and untreated DNA was small (≤ 0.5 C_t_-value). No significant difference between the two data sets was found (One Way ANOVA, *p* = 0.872, *n* = 16) and no consistent improvement in DNA template concentration was observed. A linear correlation with a slope close to one was found between the C_t_ values of the DNA treated with the Repair Mix and the C_t_ values of the untreated DNA aliquots: *y* = -0.875 + 1.037*x*, *R* ^2^ = 0.96, *n* =16, *p* < 0.001, where *x* is the C_t_ value of the untreated DNA and *y* is the C_t_ value of the treated DNA as measured by qPCR (Figure S2 in File S1). Thus, the application of the DNA Repair Mix did not lead to increased genotype abundance from the heat-desiccated samples. 

### Polymorphisms occurring in treated and untreated heat-desiccated DNA

**Figure 2:**
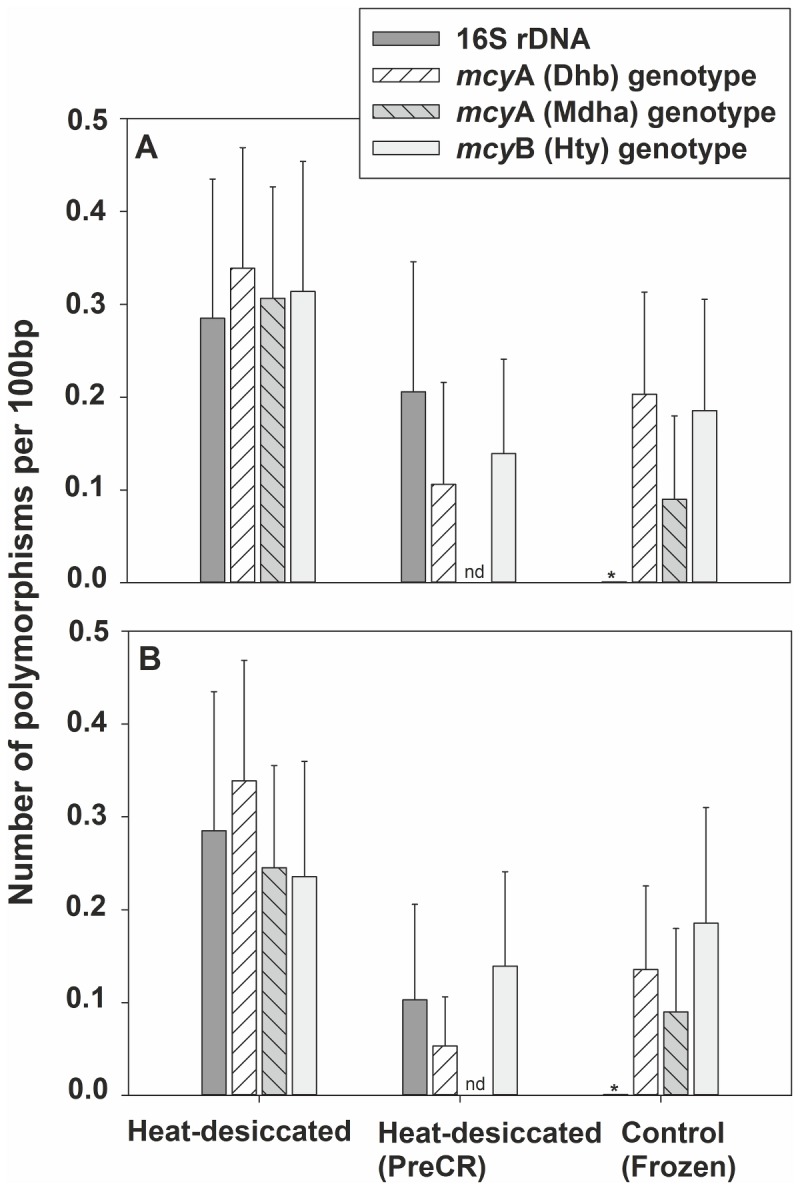
Number of substitutions found in gene loci used for qPCR and detected by sequencing from heat-desiccated DNA, an aliquot treated with the PreCR Repair Mix, and DNA from a frozen sample. (A) Number of substitutions; (B) number of substitutions in the primer sites (per 100 bp). *, no substitutions detected; nd, no data.

In order to find out whether the PreCR Repair Mix had a qualitative effect reducing the number of nucleotide substitutions, gene fragments targeted by qPCR (16S rDNA, Dhb, Mdha, and Hty) were amplified, cloned, sequenced, and compared with the sequences obtained from untreated DNA aliquots. From the sample from 1980 without treatment, 21% of the sequences (*n* = 81) showed nucleotide substitutions and in one case a nucleotide deletion. In contrast, from the treated DNA aliquot, only 12% (*n* = 57) contained nucleotide substitutions. From the frozen DNA sample, a similar proportion of polymorphisms (9%) were detected (*n* = 79), (Figure 2A). This resulted in an error rate (number of substitutions per nucleotide) of 2.5×10^-3^, 1.4×10^-3^, and 1.0×10^-3^ for the untreated (heat-desiccated) DNA, treated (heat-desiccated) DNA and frozen DNA, respectively. The same trend was observed when comparing only the binding region of the primers and the TaqMan probe: For each of the gene fragments, an increased number of substitutions were found in the heat-desiccated DNA sample (19% of the sequences, *n* = 81) when compared with the treated aliquot (9%, *n* = 57). The smallest proportion was observed from the DNA sample stored frozen (8%, *n* = 79), Figure 2B.

Overall, the highest numbers of DNA modifications were composed of transitions (87%), of which 55% were A → G and T → C changes, and 32% were C → T and G → A changes. From the untreated and the treated DNA aliquot, 82% (*n* = 17) and 86% (*n* = 7) of the substitutions were transitions, while within sequences from the DNA stored frozen only transitions were found (*n* = 7), (Table 1). The rates of total transitional substitutions per nucleotide were 2.1×10^-3^, 1.2×10^-3^, and 1.0×10^-3^ for the sequences obtained from the heat-desiccated DNA, treated heat-desiccated DNA, and frozen DNA, respectively. No transversion occurred in the sequences obtained from the frozen sample but 2% (*n* = 57) and 1% (*n* = 81) of sequences obtained from the treated and untreated DNA aliquot, respectively were affected by transversions. 

**Table 1:** Rate of nucleotide variability (per 100 bp) detected in DNA sequences obtained from heat-desiccated DNA (from 1980), an aliquot treated with the PreCR Mix, and DNA from frozen samples (from 2007).

Sample type and analyzed locus	Number of analyzed sequences	Variability in primer and probe binding sites	Variability within the total gene fragment		A→G	G→A	T→C	C→T	C→A	T→G	Gap
	16S rDNA (81 bp, 48.8% GC) ^a^										
Heat-desiccated	13	0.28	0.28		0.09	0	0	0.19	0	0	0
Heat-desiccated^+ b^	12	0.1	0.21		0	0.1	0.1	0	0	0	0
Frozen	17	0	0		0	0	0	0	0	0	0
	Dhb (*mcy*AA1, 82 bp, 37.3% GC)										
Heat-desiccated	18	0.34	0.34		0.14	0.07	0	0	0.07	0.07	0
Heat-desiccated^+^	23	0.05	0.11		0.05	0	0	0	0.05	0	0
Frozen	18	0.14	0.2		0.07	0	0.07	0.07	0	0	0
	Mdha (*mcy*AA1, 77 bp, 37.7% GC)										
Heat-desiccated	22	0.25	0.31		0.25	0	0	0	0	0	0.06
Frozen	15	0.09	0.09		0	0	0.09	0	0	0	0
	Hty (*mcy*BA1, 98 bp, 42.9% GC)										
Heat-desiccated	13	0.31	0.31		0.16	0.08	0	0.08	0	0	0
Heat-desiccated^+^	22	0.14	0.14		0	0.09	0	0.05	0	0	0
Frozen	11	0.19	0.19		0.09	0	0.09	0	0	0	0

^a^ analyzed gene locus, and length of the analyzed region (bp) and guanine – cytosine content (%) given in brackets

^b^^+^ indicates the treatment of the DNA sample with PreCR Repair Mix

Between different loci of the heat-desiccated DNA the rate of substitutions was similar: 23% of the sequences of 16S rDNA (*n* = 13), 28% of Dhb (*n* = 18), 23% of Mdha (*n* = 22), and 31% of Hty (*n* = 13). It is concluded that as a result of heat desiccation qualitative changes in DNA nucleotide sequences occurred that could be partly reverted by the PreCR Repair Mix. However, since nucleotide substitutions were evenly distributed between gene loci, the chance that DNA nucleotide sequence substitutions influence the quantification of genotype proportions by qPCR was considered negligible.

### Quantification of microcystin genotype composition in Lake Zürich

**Figure 3:**
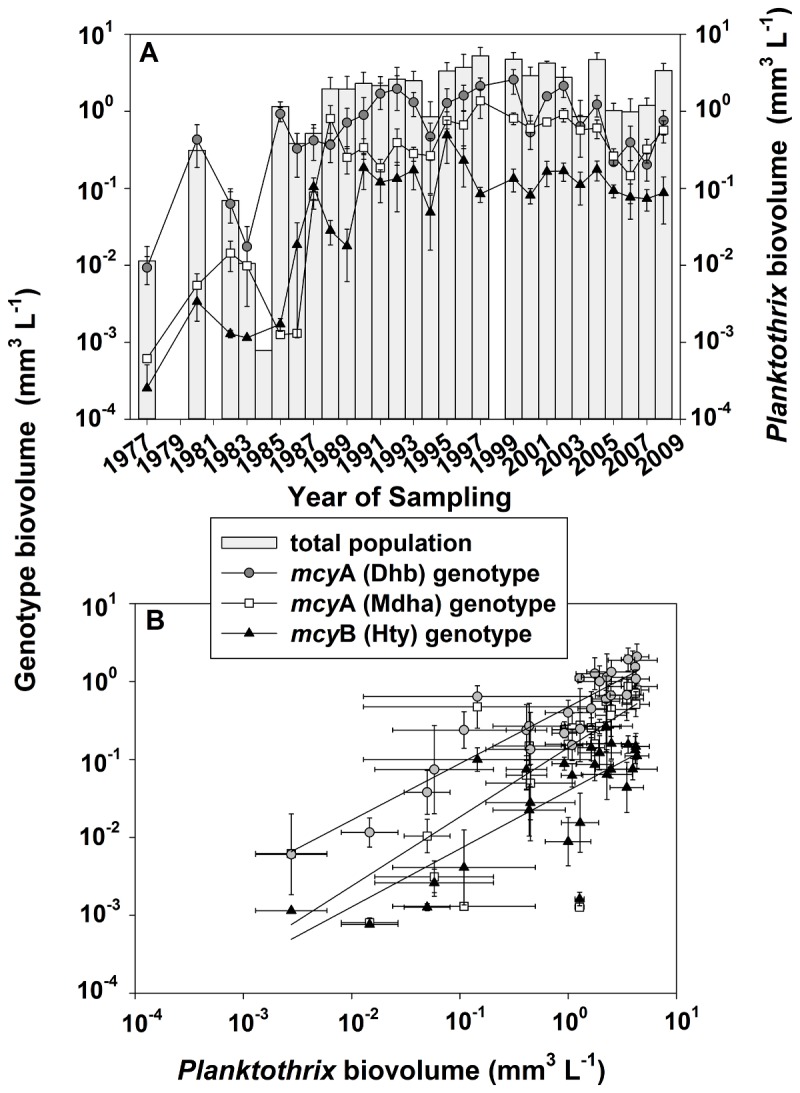
Dependence of *mcy* genotype biovolume on total population abundance as determined by qPCR in Lake Zürich. (A) Time course of annual mean ± SE abundance of the Dhb, Mdha, and Hty genotype and the total population biovolume (grey bars) of *Planktothrix* (mm^3^ L^-1^). (B) Regression curves between Dhb, Mdha, and Hty genotype and the total population. For the regression curve details, see text.

Within the period 1977–2008 the abundance of three different *Planktothrix* genotypes indicative of Dhb or Mdha in position 7 or of Hty in position 2 of the MC molecule was determined and compared with the changes in the total *Planktothrix* population density. Except for 1984, the year with the lowest *Planktothrix* biovolume, all three genotypes were detected in all the years (Figure 3A). Taking all of the samples together, 88% were positive for the Dhb, Mdha, and Hty genotype. On average, the abundance of the Dhb genotype was the highest (min 0.013, mean ± SE, 0.9 ± 0.14, max 2.6 mm^3^ biovolume L^-1^, *n* = 27), while the Mdha genotype occurred in lower concentrations only (0.001, 0.4 ± 0.3, 1.4 mm^3^ L^-1^, *n* = 27). Except for the years 1985-1987 the Hty genotype (0.0003, 0.1 ± 0.01, 0.49 mm^3^ L^-1^, *n* = 27) occurred with the lowest abundance (Table S3 in File S1).

During the study period the annual average abundance of the three different genotypes varied three (Mdha genotype), two (Dhb genotype), and three (Hty genotype) orders of magnitude and there was a highly significant linear relation of each genotype abundance with the total population density as revealed by 16S rDNA (Figure 3B, Table 2). In particular, the abundance of Dhb and Mdha genotypes were related to the total population density following the linear regression: *y* = -0.3282 + 0.725*x*, (*n* = 27, *R* ^2^ = 0.83) and *y* = -0.85+ 0.888*x*, (*n* = 27, *R* ^2^ = 0.59), where *x* is the annual average log_10_ biovolume determined by 16S rDNA (mm^3^ L^-1^) and *y* is the annual average log_10_ biovolume of the Dhb or Mdha genotype, respectively. Similarly, the relationship between the Hty genotype and the total population followed the linear regression: *y* = -1.402 + 0.745*x*, (*n* = 27, *R* ^2^ = 0.62), where *x* is the annual average log_10_ biovolume determined by 16S rDNA (mm^3^ L^-1^) and *y* is the annual average log_10_ biovolume of the Hty genotype. The linear regressions differed in intercept but not in slope (One Way ANOVA, group effect: *p* < 0.001, interaction group × covariate: *p* > 0.3).

**Table 2:** Correlation coefficients between biovolume (mm^3^ L^-1^) and the proportion of the Dhb, Mdha, and Hty genotypes and the genotype carrying the *mcy*HA deletion (data from [19]).

	Total population (mm^3^ L^-1^)	Dhb (mm^3^ L^-1^)	Dhb (%)	Mdha (mm^3^ L^-1^)	Mdha (%)	Hty (mm^3^ L^-1^)	Hty (%)	Del (mm^3^ L^-1^)	Del%
Total population (mm^3^ L^-1^)	-								
Dhb (mm^3^ L^-1^)	0.61**	-							
Dhb (%)	-0.31	-0.08	-						
Mdha (mm^3^ L^-1^)	0.58**	0.7**	-0.48*	-					
Mdha (%)	0.12	-0.14	-0.21	0.27	-				
Hty (mm^3^ L^-1^)	0.27	0.5**	-0.37	**0.48**	0.04	-			
Hty (%)	-0.23	-0.15	0.1	-0.17	0.17	**0.44**	-		
*mcy*HA-Del (mm^3^ L^-1^)	0.35	**0.4**	-0.66**	0.71**	0.19	**0.42**	-0.2	-	
*mcy*HA-Del (%)	0.26	0.23	-0.6**	**0.48**	**0.41**	0.39	-0.14	0.79**	-

Significant values are marked in bold *p* < 0.05, * *p* < 0.01, ** *p* < 0.001.

**Figure 4:**
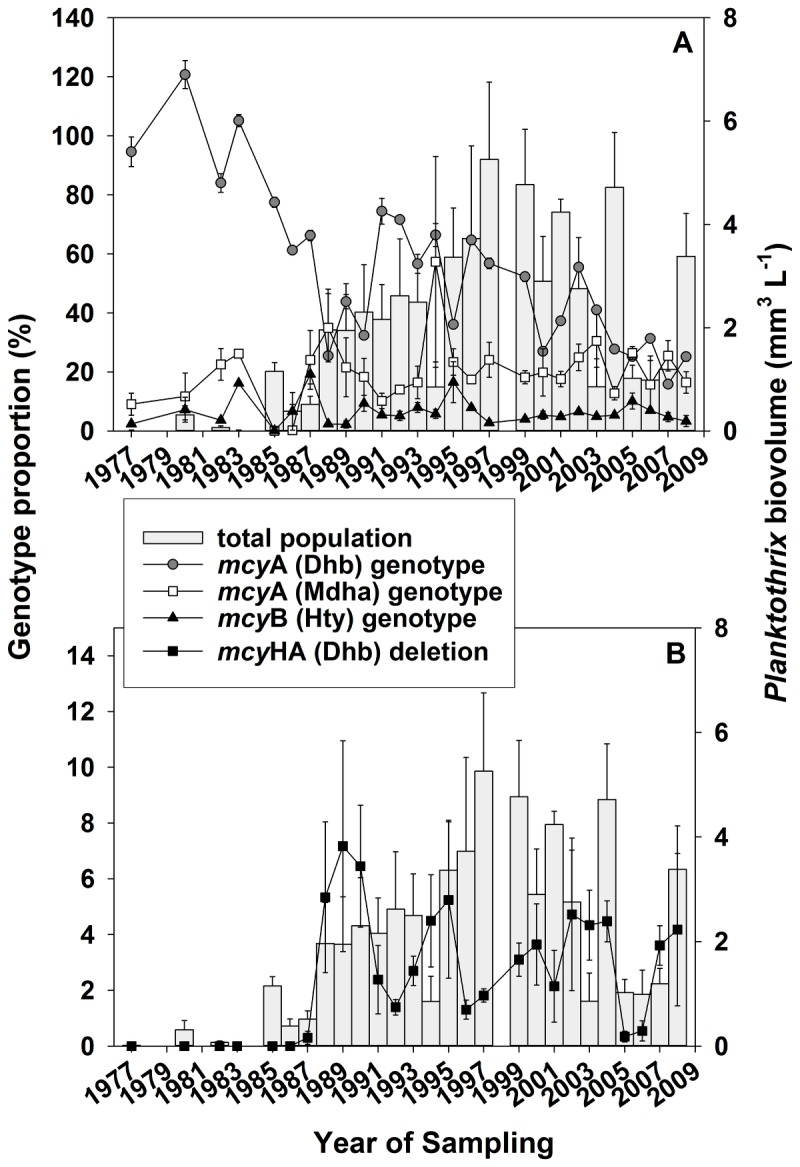
Proportion of individual genotypes as determined by qPCR in Lake Zürich. (A) Time course of annual mean ± SE proportion of the Dhb, Mdha, and Hty genotype and (B) of the *mcy*HA deletion genotype and the total population biovolume (grey bars) of *Planktothrix* (mm^3^ L^-1^).

Consequently, there was a relatively high stability in overall *mcy* genotype composition over the study period. With the exception of year 2007, the Dhb genotype occurred in the highest proportion (min, mean ± SE, max, 16%, 55 ± 5%, 141% of the total population, *n* = 27). On average, the Mdha genotype occurred in lower proportion (0.1%, 20 ± 2%, 57.3%). The Hty genotype occurred in the lowest proportion (0.1%, 19.3 ± 0.9%, 6.6%), Figure 4A. Nevertheless, over the study period, the Dhb genotype decreased in its proportion from the first decade (1977-1989: mean ± SE, 76 ± 11%, *n* = 9) to the two subsequent decades (1990–1999: 57 ± 5%, *n* = 9; 2000–2008: 32 ± 4%, *n* = 9), *p* < 0.01 (Kruskal-Wallis One Way ANOVA). Unexpectedly, for the Mdha genotype proportion no corresponding increase from the first decade (1977-1989: 17 ± 4%, *n* = 9) to the two subsequent decades was found (1990-1999: 22 ± 5%, *n* = 9, 2000-2008: mean 22 ± 2%, *n* = 9, *p* = 0.49). The lowest Hty genotype proportion was found to be stable between the decades (1977-1989: mean 7 ± 2%, *n* = 9, 1990-1999: mean 7 ± 1%, *n* = 9, 2000-2008: mean 6 ± 0.6%, *n* = 9, *p* = 0.47), Table S3 in File S1.

In order to explain the decline of the Dhb genotype, each of the *mcy*A/B genotypes was compared in abundance to the only genotype carrying a 1.8 kbp deletion of the Dhb gene [19]. As this 1.8 kbp deletion ranged from part of *mcy*H to *mcy*A it prevented the amplification of the Dhb genotype by qPCR [21,24]. From the year 1987 onwards the *mcy*HA (Dhb) deletion genotype occurred consistently and showed a significant relation in its abundance with the total population density (Table 2, Figure 4B). From 1987 onwards the proportion of the *mcy*HA deletion mutant varied from 0.3–7.2% (mean ± SE, 3.3% ± 0.4). Only the proportion of the Dhb genotype was significantly negatively related with the *mcy*HA deletion genotype biovolume as well as its proportion (Table 2). In contrast, the proportions of the Mdha and Hty genotypes were not related to the biovolume of the *mcy*HA deletion genotype. Consequently, it is concluded that part of the decline of the Dhb genotype proportion that occurred since 1987 could be related to its *mcy*HA deletion mutant. The overall relationship between the three different *mcy* genotypes, however, was stable and showed a minor change only when compared with the more than 5,000–fold increase (min 0.001, max 5.3 mm^3^ L^-1^) of the total population.

## Discussion

### DNA quality isolated from heat-desiccated samples

In this study, the most common substitutions were transitions, i.e. the highest number of these was A → G and T → C or C → T and G → A conversions. The rate of these substitutions was higher compared to the DNA isolated from frozen samples (Figure 2). In studies investigating ancient DNA, nucleotide substitutions are commonly observed, specifically transitions A → G or T → C and C → T and G → A [25]. It has been shown that elevated temperatures promote the hydrolytic deamination of adenine to hypoxanthine [26] and the conversion of cytosine to uracil in DNA [27]. Lindahl and Nyberg [27] estimated that in DNA incubated at 100°C (10 min), one uracil residue per 5,000 cytosine residues will be generated. In addition, Bruskov et al. [28] showed the heat-mediated oxidation of guanine to 8-oxoguanine, which can pair with adenine. Karran and Lindahl [26] showed that the rate of deamination of adenine is a rather slow process compared to the deamination of cytosine, which occurs at a 40-fold faster rate in DNA heated to 110°C. Since in this study the phytoplankton biomass used for DNA isolation was dried at 110°C (2 h) it was reasonable to expect that the PreCR Repair Mix used to reverse the high-temperature induced substitutions resulted in a significant increase in genotype abundance as estimated by qPCR. However, this was not the case (Figure S2 in File S1). Furthermore, no improvement in the detectability of rare inactive *Planktothrix* genotypes such as the *mcy*HA deletion mutant [24] could be achieved after the treatment of the heat-desiccated DNA with the PreCR Repair Mix compared to untreated DNA (data not shown). Nevertheless, the activity of the PreCR Repair enzyme mix was demonstrated when comparing the number of nucleotide substitutions between the heat-desiccated DNA and the treated aliquot. The 6.6-fold reduced occurrence of A → G nucleotide substitutions and 1.7-fold reduced occurrence of all the transitions found in the treated DNA when compared with the untreated DNA complies with the enzymatic reversion of e.g. uracil to cytosine or the repair of abasic sites. In total, the observed nucleotide substitutions occurred with a frequency far too low (~ 10^-3^) implying that those errors could not account for the observed reduction in DNA template concentration. Instead we found the heat-desiccated DNA to be highly fragmented, which is in accordance to earlier studies [29]. It is concluded that through the high temperature, rather the fragmentation of DNA into small fragments, than the heat-induced substitutions was the reason for decreased DNA quality.

### Abundance of individual microcystin genotypes

Over a study period of thirty years, a highly significant positive correlation was found between the abundance of two *mcy*A genotypes and one *mcy*B genotype and the total *Planktothrix* population density. While such a relationship has been found when comparing *mcy* genotype composition between lakes [17], this study shows that even during a thirty-year period of re-oligotrophication and a population increase by three orders of magnitude the *mcy* genotype composition was stable. This implies that the individual *mcy* genotype subpopulations constitute a stable part of the total population and are not likely to disappear or reappear within short time periods. 

Nevertheless, a decrease in the proportion of the Dhb genotype was observed between the first and the following two decades (Figure 4A, B). In contrast, the Mdha genotype did not show a corresponding increase in abundance, suggesting that a significant part of the *Planktothrix* population remained undetected by means of qPCR. A natural factor that may explain a possible decrease in the proportion of the Dhb genotype over time would be the parallel increase of an unknown genotype affected by mutation within the gene region encoding for the first A-domain of the *mcy*A gene. One such genotype that was described earlier showed a 1.8 kbp deletion within *mcy*HA affecting the Dhb primer binding region and thus prevented detection by qPCR [24]. Overall, the proportion of this genotype was rather low (Figure 4) and as such cannot explain the decrease of the Dhb genotype during the last decade completely. So far, we cannot exclude additional (unknown) *mcy*A deletion genotypes that occurred in Lake Zürich. For example, due to the accuracy of the TaqMan Assay, even *mcy*HA deletion mutants carrying single substitutions would have led to a reduced detectability.

However, when compared with the decline of the Dhb genotype proportion over time, the proportion of its *mcy*HA deletion mutant clearly showed the opposite trend (Figure 4). Furthermore, the abundance of the *mcy*HA deletion mutant was positively related with the total *Planktothrix* population density. Among twelve lakes in the Alps (Austria, Germany, Switzerland) that were sampled during 2005-2007 [24], an overall increase of the abundance of mutants carrying either the *mcy*HA deletion or insertion of transposable elements with the total *Planktothrix* population density has been observed. Consequently, it is reasonable to conclude that the abundance of mutations in the *mcy* gene cluster and in particular the abundance of the *mcy*HA genotype is generally favored by the total population density.

On the other hand, the evidence that also the proportion of the *mcy*HA deletion mutant is favored by an increase in total population density is scarce. In the same study [24], when comparing sparse and dense *Planktothrix* populations among twelve lakes of the Alps it could be shown that the same mutations that were absent from depth-integrated water samples could be found when using enriched plankton net samples (Figure 3 in [24]). Through the observation period of this study, the decrease of the Dhb proportion from the 1980s to the 1990s indirectly could also show a proportional increase of the *mcy*HA deletion. While *Microcystis* strains, which contained deletions of several *mcy* genes, have been observed to outgrow the original strain under culture conditions [30,31], those observations so far could not be made under field conditions. No further increase of the *mcy*HA mutant since its first detection in 1987 until 2008 was observed. Consequently, it is likely that the proportional increase of the *mcy*HA deletion genotype in lakes of the Alps is rather slow, requiring even longer observation periods than applied in this study. The reasons for this overall slow increase are possibly selective pressure preventing a further increase, for example through grazing or parasitism [19]. Another possible explanation is that through the deletion of part of the *mcy*HA genes the advantage in growth rate resulting from the release from the energetic costs involved in MC biosynthesis is too small to become relevant. 

### Potential microcystin biosynthesis

In this study, the average abundance of the Dhb genotype responsible for the incorporation of Dhb in position 7 of the MC molecule was significantly higher when compared with the Mdha genotype responsible for the incorporation of Mdha in position 7 of the MC molecule. Recently, we could show that the abundance and proportion of the Dhb or Mdha genotypes was significantly related to the concentration of the respective MC structural variants as revealed by HPLC [17]. Frequently, the MCs isolated from samples of red-pigmented *P. rubescens* were dominated by MCs carrying Dhb in position 7 ([D-Asp^3^, Dhb^7^]-MC-RR: in Lake Steinsfjorden (Norway) [32], in Hallwiler See (Switzerland) [33], in Lac du Bourget (France) [34], in Talsperre Weida, Talsperre Pöhl, Arendsee, Germany [35]. On the other hand, Ernst et al. [36] reported higher amounts of [D-Asp^3^]-MC-RR compared with [D-Asp^3^, Dhb^7^]-MC-RR from *P. rubescens* in Lake Ammersee (Germany) during 1999–2004. Correspondingly, a survey of lakes of the Alps from 2005 until 2007 [17] showed that the average proportion of the Mdha genotype exceeded the Dhb genotype proportion in Lake Ammersee resulting in the dominance of [D-Asp^3^, Mdha^7^]-MC-RR and a minor proportion of [D-Asp^3^, Dhb^7^]-MC-RR [17]. It is not known as to whether this spatial variation in Dhb vs Mdha in position 7 of the MC molecule among spatially isolated *P. rubescens* populations is of adaptive significance. With regard to the much more pronounced structural variation of variable amino acids in position 2 of the MC molecule a similar distinct spatial variation has been reported [37]. Earlier, from comparing the genetic information from the A-domains of isolated *Planktothrix* strains, it was concluded that the genetic variation could imply either the relaxation of selective constrains or adaptive significance [37]. However, from the population genetic structure as recorded directly from field samples it could be shown that populations in different habitats were sufficiently isolated to allow a random genetic drift to occur. As observed during this study, the results on the rather stable genetic population structure over three decades support this hypothesis.

The increase in the frequency of the *mcy*A (Dhb) deletion mutant during the 30 year observation period is of significance with regard to the overall MC net production of the population in Lake Zürich. During the earlier survey on *mcy* mutant genotype abundance [24], a total of 103-114 individual filaments of *Planktothrix* were isolated from a depth-integrated sample from each of the twelve lakes and analyzed for MC production by means of matrix assisted laser desorption ionization-time of flight mass spectrometry [38]. As a result from a total of 108 filaments isolated from Lake Zürich (20 January 2006) only six filaments did not show MC. Thus, the proportion of inactive *mcy* genotypes (5.6%) compares with the range in the proportion of the *mcy*HA deletion mutant (Figure 4B, 0.3-7.2%). Consequently, so far the increase of the *mcy*HA gene deletion mutant has had a minor effect on the overall toxicity of the *Planktothrix* population in L. Zürich. 

## Supporting Information

File S1 **Supporting figures and tables.**
Table S1. Oligonucleotide primers used to amplify the target regions for qPCR from heat-desiccated DNA samples. Table S2. Influence of heat-desiccation of Planktothrix cells on qPCR quantification. Table S3. Average ± SE biovolume per year of the total Planktothrix population, and biovolume and proportions of mcy genotypes in Lake Zürich between 1977 and 2008. Figure S1. DNA isolated from heat-desiccated vs. frozen phytoplankton samples obtained from Lake Zürich. Figure S2. Ct values (mean ± SE) as determined by qPCR using four different gene loci of Planktothrix (16S rDNA, Dhb, Mdha and Hty genotype) from heat-desiccated DNA and treated heat-desiccated DNA.
(DOCX)
