# Supplementary material for: Genetic Variability of Microcystin Biosynthesis Genes in Planktothrix as Elucidated from Samples Preserved by Heat Desiccation during Three Decades
Source: PLoS One. 2013 Nov 12;8(11):e80177. doi: 10.1371/journal.pone.0080177 (PMC3827215; doi:10.1371/journal.pone.0080177)
Supplement: File S1 — Supporting figures and tables. Table S1. Oligonucleotide primers used to amplify the target regions for qPCR from heat-desiccated DNA samples. Table S2. Influence of heat-desiccation of Planktothrix cells on qPCR quantification. Table S3. Average ± SE biovolume per year of the total Planktothrix population, and biovolume and proportions of mcy genotypes in Lake Zürich between 1977 and 2008. Figure S1. DNA isolated from heat-desiccated vs. frozen phytoplankton samples obtained from Lake Zürich. Figure S2. Ct values (mean ± SE) as determined by qPCR using four different gene loci of Planktothrix (16S rDNA, Dhb, Mdha and Hty genotype) from heat-desiccated DNA and treated heat-desiccated DNA. (DOCX) [file pone.0080177.s001.docx]

### Table S1 Oligonucleotide primers used to amplify the target regions for qPCR from heat-desiccated DNA samples. The primer pairs were designed to amplify the whole qPCR targeted gene fragment and subsequently the PCR products were cloned and sequenced to detect heat-induced DNA damages.

| Application | Target region | Forward primer / reverse primer (5'-3') | Annealing T (°C) | Elongation time (s) | Amplicon length (bp) |
| --- | --- | --- | --- | --- | --- |
|  |  |  |  |  |  |
| Amplification of gene fragments targeted by qPCR | | |  |  |  |
|  | 16S rDNA | ggcgtaaagagtccgtaggtagt / ttcaccgctacaccaggaat | 60 | 12 | 129 |
|  | Dhb | ttaatcttaactcaagaaaaattagtcgagt / gataatatgttaaattatcgggtttagcc | 60 | 12 | 143 |
|  | Mdha | gcagatacccaagtcaaaatattactca / caaaattgtctggattttcctttg | 61 | 12 | 134 |
|  | Hty | acctgctggcaatgtccac / ccgccaatatgtaattcgcc | 62 | 12 | 139 |
|  |  |  |  |  |  |

### Table S2 Influence of heat-desiccation of *Planktothrix* cells on qPCR quantification. Cell aliquots of strains PCC 7821 and No.40 were desiccated at different temperatures (80, 110 and 150°C) for two and twelve hours, respectively. As a control aliquots of the strains were stored frozen (-20°C). DNA was quantified by qPCR with regard to mean ± SE biovolume (16S rDNA) and the influence on mean ± SE proportions of Dhb and Mdha genotypes. The experiment was repeated twice. P-values for testing treatment effects (factor °C, factor h) were estimated by ANOVA (*n* = 21).

| Strain | Treatment | | 16S rDNA (mm^3^L^-1^) | Dhb or Mdha genotype (%) |
| --- | --- | --- | --- | --- |
| Dhb genotype (PCC 7821) | |  |  |  |
|  | 80°C (2h) | | 48 ± 32^ab^ | 78 ± 10 |
|  | 110°C (2h) | | 123 ± 72^ab^ | 60 ± 34 |
|  | 150°C (2h) | | 15 ± 10^ab^ | 70 ± 15 |
|  | 80°C (12h) | | 18 ± 7^ab^ | 109 ± 27 |
|  | 110°C (12h) | | 52 ± 22^ab^ | 61 ± 34 |
|  | 150°C (12h) | | 2± 0.5^b^ | 65 ± 25 |
|  | Control | | 242 ± 50^a^ | 38 ± 4 |
|  | Factor °C | | *P* < 0.001 | *P* = 0.12 |
|  | Factor h | | *P* < 0.001 | *P* = 0.33 |
|  | °C × h | | *P* = 0.72 | *P* = 0.29 |
| Mdha genotype (No. 40) | |  |  |  |
|  | 80°C (2h) | | 40 ± 15^ab^ | 68 ± 13 |
|  | 110°C (2h) | | 50 ± 22^ab^ | 46 ± 6 |
|  | 150°C (2h) | | 32 ± 19^ab^ | 41 ± 2 |
|  | 80°C (12h) | | 28 ± 16^ab^ | 55 ± 12 |
|  | 110°C (12h) | | 37 ± 6^ab^ | 46 ± 9 |
|  | 150°C (12h) | | 0.8 ± 0.2^b^ | 53 ± 20 |
|  | Control | | 369 ± 57^a^ | 44 ± 3 |
|  | Factor °C | | *P* < 0.001 | *P* = 0.4 |
|  | Factor h | | *P* < 0.001 | *P* = 0.84 |
|  | °C × h | | *P* = 0.91 | *P* = 0.53 |

**Table S3** Average ± SE biovolume per year of the total *Planktothrix* population, and biovolume and proportions of *mcy* genotypes in Lake Zürich between 1977 and 2008 as estimated by qPCR. Biovolume in mm^3^ L^-1^, genotype proportions in percentage of the total population as estimated by 16S rDNA.

| Year | 16S rDNA | Dhb genotype | | Mdha genotype | | Hty genotype | | *mcy*HA deletion genotype | | |
| --- | --- | --- | --- | --- | --- | --- | --- | --- | --- | --- |
|  | mm^3^ L^-1^ | mm^3^ L^-1^ | % | mm^3^ L^-1^ | % | mm^3^ L^-1^ | % | mm^3^ L^-1^ | % |  |
| 1977 | 0.02 ± 0.01 | 0.01 ± 0.01 | 80 ± 12 | 0.001 ± 0.0001 | 7 ±2 | 0.0003 ± 0.0003 | 2.4 | nd |  |  |
| 1980 | 0.27 ± 0.16 | 0.37 ± 0.2 | 142 ± 33 | 0.004 ± 0.002 | 15 ± 11 | 0.003 ±0.001 | 7.2 ± 4.3 | nd |  |  |
| 1982 | 0.07 ± 0.03 | 0.06 ± 0.03 | 84 ± 18 | 0.014 ± 0.01 | 23 ± 5 | 0.001 ± 0.0001 | 3.7 ± 0.8 | nd |  |  |
| 1983 | 0.01 ± 0.01 | 0.01 ± 0.01 | 94 ± 15 | 0.007 | 24 | 0.001 | 16 | nd |  |  |
| 1984 | 0.0008 ± 0.0002 | nd |  | nd |  | nd |  | nd |  |  |
| 1985 | 1.31 ± 0.12 | 1.11 ± 0.07 | 86 ± 4 | 0.001 ± 3×10^-5^ | 0.1 ± 0.01 | 0.002 ± 0.0003 | 0.1 ± 0.02 | nd |  |  |
| 1986 | 0.38 ± 0.14 | 0.33 ± 0.19 | 61 ± 31 | 0.001 ± 0.0001 | 0.26 ± 0.03 | 0.02 ± 0.02 | 6.7 ± 6.4 | nd |  |  |
| 1987 | 0.52 ± 0.16 | 0.42 ± 0.19 | 66 ± 18 | 0.08 ± 0.03 | 24 ± 10 | 0.1 ± 0.03 | 19.3 ± 3.3 | 0.0005 ± 0.0003 | 0.3 ±0.2 |  |
| 1988 | 1.62 ± 0.54 | 0.35 ± 0.16 | 26 ± 9 | 0.65 ± 0.3 | 35 ± 12 | 0.03 ± 0.01 | 2.4 ± 0.6 | 0.08 ± 0.05 | 5.3 ± 2.7 |  |
| 1989 | 1.36 ± 053 | 0.52 ± 0.23 | 45 ± 13 | 0.22 ± 0.1 | 22 ± 10 | 0.02 ± 0.01 | 2.2 ± 1.2 | 0.06 ± 0.03 | 7.2 ± 3.8 |  |
| 1990 | 1.94 ± 0.57 | 0.74 ± 0.5 | 34 ± 12 | 0.29 ± 0.1 | 18 ± 6 | 0.19 ± 0.1 | 9.4 ± 2.7 | 0.12 ± 0.05 | 6.5 ± 2.2 |  |
| 1991 | 2.16 ± 0.7 | 1.69 ± 0.6 | 75 ± 12 | 0.19 ± 0.1 | 10 ± 3 | 0.12 ± 0.1 | 5.4 ± 0.9 | 0.03 ± 0.01 | 2.4 ± 1.3 |  |
| 1992 | 2.5 ± 1.1 | 1.96 ± 0.9 | 72 ± 19 | 0.4 ± 0.4 | 14 ± 1.3 | 0.13 ± 0.1 | 5.2 ± 1.6 | 0.03 ± 0.01 | 1.4 ± 0.3 |  |
| 1993 | 2.5 ± 0.8 | 1.31 ± 0.5 | 57 ± 12 | 0.28 ± 0.1 | 17 ± 6 | 0.17 ± 0.1 | 8.0 ± 1.7 | 0.05 ± 0.01 | 2.7 ± 0.5 |  |
| 1994 | 0.85 ± 0.5 | 0.5 ± 0.2 | 66 ± 14 | 0.27 ± 0.2 | 57 ± 36 | 0.05 ± 0.03 | 5.8 ± 1.5 | 0.04 ± 0.03 | 4.5 ± 1.7 |  |
| 1995 | 2.6 ± 0.7 | 1.1 ± 0.5 | 39 ± 16 | 0.65 ± 0.2 | 26 ± 3 | 0.49 ± 0.3 | 16.5 ± 7 | 0.08 ± 0.01 | 5.2 ± 2.8 |  |
| 1996 | 3.73 ± 1.8 | 1.6 ± 0.6 | 65 ± 20 | 0.67 ± 0.5 | 18 ± 0.5 | 0.23 ± 0.1 | 7.9 ± 0.8 | 0.04 ± 0.02 | 1.3 ± 0.3 |  |
| 1997 | 5.26 ± 1.5 | 2.13 ± 0.6 | 57 ± 20 | 1.37 ± 0.8 | 24 ± 6 | 0.08 ± 0.02 | 2.8 ± 0.9 | 0.08 ± 0.03 | 1.8 ± 0.2 |  |
| 1999 | 4.8 ±1.1 | 2.58 ± 0.9 | 52 ± 11 | 0.82 ± 0.1 | 18 ± 2 | 0.13 ± 0.04 | 4 ± 0.2 | 0.09 ± 0.02 | 3.1 ± 0.6 |  |
| 2000 | 2.9 ± 0.9 | 0.71 ± 0.2 | 27 ± 5 | 0.6 ± 0.4 | 20 ± 8 | 0.08 ± 0.02 | 5.4 ± 1.4 | 0.11 ± 0.07 | 3.6 ± 1.5 |  |
| 2001 | 4.24 ± 0.3 | 1.56 ± 0.2 | 37 ± 5 | 0.73 ± 0.1 | 18 ± 3 | 0.16 ± 0.1 | 4.9 ± 1 | 0.07 ± 0.05 | 2.1 ± 1.3 |  |
| 2002 | 3.67 ± 0.5 | 2.13 ± 0.6 | 56 ± 10 | 0.91 ± 0.3 | 25 ± 5 | 0.17 ± 0.04 | 6.5 ± 0.6 | 0.1 ± 0.08 | 4.7 ± 2.7 |  |
| 2003 | 1.14 ± 0.7 | 0.64 ± 0.02 | 41 ± 12 | 0.57 ± 0.3 | 31 ± 9 | 0.11 ± 0.1 | 4.9 ± 0.3 | 0.09 ± 0.02 | 4.3 ± 1.3 |  |
| 2004 | 4.72 ± 1.1 | 1.22 ± 0.4 | 28 ± 6 | 0.61 ± 0.2 | 13 ± 2 | 0.18 ± 0.1 | 5.4 ± 0.6 | 0.1 ± 0.04 | 4.5 ± 0.7 |  |
| 2005 | 1.02 ± 0.3 | 0.22 ± 0.01 | 25 ± 5 | 0.26 ± 0.1 | 27 ± 2 | 0.09 ± 0.02 | 10.2 ± 2.7 | 0.003 ± 0.002 | 0.34 ± 0.2 |  |
| 2006 | 0.99 ± 0.5 | 0.39 ± 0.3 | 31 ± 6 | 0.15 ± 0.1 | 16± 8 | 0.08 ± 0.04 | 7 ± 0.6 | 0.01 ± 0.01 | 0.5 ± 0.4 |  |
| 2007 | 1.19 ± 0.3 | 0.2 ± 0.1 | 16 ± 5 | 0.32 ± 0.2 | 26 ± 5 | 0.07 ± 0.02 | 4.7 ± 1.5 | 0.07 ± 0.04 | 3.6 ± 0.7 |  |
| 2008 | 4.26 ± 1.6 | 1.29 ± 0.5 | 28 ± 8 | 1.1 ± 0.9 | 20 ± 6 | 0.09 ± 0.1 | 3.4 ± 1.9 | 0.1 ± 0.08 | 4.2 ± 2.7 |  |

nd, not detected

A B C D E F G H J K L M N O P

**Figure S1** DNA isolated from heat-desiccated vs. frozen phytoplankton samples obtained from Lake Zürich. Samples from the years 1980, 1985, 1990, 1995 (heat-desiccated) and 2005, 2006 (frozen) are shown. Lane A: Gene Ruler DNA Ladder, Ultra Low Range (Fermentas), Lanes B, C and E: DNA from 1980, lanes F, G: DNA from 1985, lanes D, K and L: 1990, lanes H, J: 1995, lanes M, N: 2005 and lane O: 2006, lane P: ƛ PstI DNA marker.

**Figure S2** C_t_ values (mean ± SE) as determined by qPCR using four different gene loci of *Planktothrix* (16S rDNA, Dhb, Mdha and Hty genotype) from heat-desiccated DNA and treated heat-desiccated DNA. The aliquots of the heat-desiccated untreated DNA were measured twice to control for random errors and high reproducibility was found between repeated measurements (data not shown).
